# Blood Platelets

**Published:** 1914-06

**Authors:** 


**Blood Platelets.** Gerald B. Webb, G. Burton Gilbert, and Leon Havens, of Cragmore Sanitarium, Colorado Medicine, January, 1914; allude to previous studies corroborated by others, showing that high altitude causes an increase of lymphacytes, known to be antagonistic to the tubercle bacillus, the polymorphonuclears diminishing. An increase in the plaques also occurs. From experiments on rabbits, they also conclude that the plaques are increased in carbon monoxid poisoning, hyperaemia of the marrow of long bones and in tuberculosis. Observations of human patients confirm the last, for all stages of tuberculosis of the lungs and they believe that the plaques are a source of opsonins.

